# Innate and Adaptive Immunity Synergize to Trigger Inflammation in the Mammary Gland

**DOI:** 10.1371/journal.pone.0154172

**Published:** 2016-04-21

**Authors:** Pascal Rainard, Patricia Cunha, Florence B. Gilbert

**Affiliations:** ISP, INRA, Université Tours, Nouzilly, France; International Centre for Genetic Engineering and Biotechnology, ITALY

## Abstract

The mammary gland is able to detect and react to bacterial intrusion through innate immunity mechanisms, but mammary inflammation can also result from antigen-specific adaptive immunity. We postulated that innate and adaptive immune responses could synergize to trigger inflammation in the mammary gland. To test this hypothesis, we immunized cows with the model antigen ovalbumin and challenged the sensitized animals with either *Escherichia coli* lipopolysaccharide (LPS) as innate immunity agonist, ovalbumin as adaptive immunity agonist, or both agonists in three different udder quarters of lactating cows. There was a significant amplification of the initial milk leukocytosis in the quarters challenged with the two agonists compared to leukocytosis in quarters challenged with LPS or ovalbumin alone. This synergistic response occurred only with the cows that developed the ovalbumin-specific inflammatory response, and there were significant correlations between milk leukocytosis and production of IL-17A and IFN-γ in a whole-blood ovalbumin stimulation assay. The antigen-specific response induced substantial concentrations of IL-17A and IFN-γ in milk contrary to the response to LPS. Such a synergy at the onset of the reaction of the mammary gland suggests that induction of antigen-specific immune response with bacterial antigens could improve the initial immune response to infection, hence reducing the bacterial load and contributing to protection.

## Introduction

Mastitis is the most costly infectious disease in dairy cows [1]. Vaccination against the main mastitis-causing pathogens would help control the disease, but few commercial vaccines are available, and their efficacy is in need of improvement [2, 3]. The most effective protection against infections usually involves integrated responses of the innate and adaptive arms of immunity. Type 17 immune defenses, based on the production of IL-17, rest on such integrated responses. At barrier sites such as the skin, respiratory, intestinal and urogenital tracts, production of IL-17 is important in host defense against extracellular bacteria mainly through the recruitment of neutrophils and its effects on epithelial cells [4]. IL-17A is produced by several cell types, including helper T cells that are high producers of this cytokine, and for this reason have been dubbed Th17 cells [5]. As a critical mediator in the coordination of host defense at barrier epithelial sites, IL-17A secreted by Th17 cells bridges innate and adaptive immunity [6]. Because infections of the mammary gland (MG) involve extracellular bacteria at an epithelium barrier, type 17 immunity appears to be an appropriate immune response. The dearth of the necessary tools and reagents has hampered investigations on type 17 immunity in cattle, but the situation is improving. A growing number of observations suggest that IL-17 and IL-17-producing cells are involved in the defense of the MG against infection.

IL-17A is produced or its encoding gene overexpressed in the bovine MG during mastitis caused by *Escherichia coli*, *Streptococcus uberis* or *Staphyloccus aureus* [7–10]. Mammary epithelial cells (MEC) were shown to respond to IL-17A and IL-17F by upregulating genes encoding chemokines and antimicrobial peptides [11]. By using mouse mastitis models, we and others showed that IL-17A is involved in the innate immune response of the MG to *S*. *aureus* infection [12, 13]. Taken together these findings indicate that IL-17A contributes to the innate immune defenses of the MG against infection by pyogenic bacteria. Regarding the adaptive arm of immunity, it has been shown that the recruitment of leucocytes into the lumen of the MG can be induced by injecting a few micrograms of a protein antigen through the teat canal of cows previously sensitized by a systemic immunization with the antigen, whereas control unimmunized cows did not react [14, 15]. During the first few hours of the reaction, more than 80% of the migrated leucocytes are neutrophils, this proportion declining gradually to about 70% in a few days [15–17]. We have dubbed this neutrophilic inflammation mammary antigen-specific reaction (mASR) [17]. We have shown that a Th17 cytokine signature is manifest in the bovine mammary gland during the mASR induced with the model antigen ovalbumin and that it is related to the production of IL-17A and INF-γ by bovine CD4+ T lymphocytes [18]. In vitro studies revealed that there is a synergistic effect exerted by IL-17A and certain Microbial-Associated Molecular Patterns (MAMPs) or live bacteria on the defense responses of mammary epithelial cells [7, 11]. This raises the question of an in vivo parallel with the in vitro synergy between IL-17A and MAMPs in the stimulation of MEC. Such a synergy would have implications in terms of modulation of the reactivity of the MG to infection by vaccination or by a previous infection. In case the resulting inflammatory response is efficient at killing the pathogen, the synergy would be desirable. If inefficient, it could be detrimental by increasing tissue damages. It is thus of interest to better characterize the cooperation between innate and adaptive immunity in order to devise means favoring or preventing the occurrence of type 17 immune responses.

The main objective of this study was to investigate whether a protein antigen and a MAMP could synergize in the induction of mammary inflammation. To test this hypothesis, we immunized cows with the model antigen ovalbumin before intramammary challenge with either ovalbumin, *E*. *coli* lipopolysaccharide (LPS) or both stimuli in three different udder quarters of the immunized cows. The subsequent milk leukocytosis was monitored to reveal the potential amplification of LPS-induced cell recruitment by sensitization to ovalbumin, and milk somatic cell counts (SCC) were correlated with the production of IL-17A and IFN-γ in an ovalbumin-specific whole blood stimulation assay.

## Materials and Methods

### Ethics Statement

All animal experiments were performed with approval from the Comité d’éthique pour l’expérimentation animale du Val de Loire (CEEA VdL), protocol number 2012-10-12 V2. Animal studies were compliant with all applicable provisions established by the European directive 2010/63/UE.

### Animals and experimental design

Lactating cows were in their first or second lactations. They had at least three healthy quarters, defined as quarters that did not shed bacteria in milk (no detectable bacterial growth in 50 μL milk samples) and with somatic cell counts (SCC) less than 150 000 cells/mL milk. In most cows the four quarters were devoid of bacteria, but the shedding of coagulase-negative staphylococci from one quarter was tolerated when not accompanied by clinical signs (sub-clinical infection). Cows with clinical mastitis were excluded from the selection.

A total of 17 Holstein female bovine animals was used in the experiments, of which 13 were challenged through the teat canal. They were maintained in their farm (teaching and research herd of the LEGTA, Vendôme, France, 47.798947N, 1.092002E; Owner and personnel consent was obtained with complete information on the protocol) during the experiments, and remained in the herd after completion of the study. They were not isolated from their herdmates during the experiment. All cows were milked two times daily and were housed in a loose-housing cowshed. They were fed a diet of hay, silage and concentrate. Intramammary injection of antigen, LPS or both induced either a subclinical inflammation or a clinical mastitis of moderate intensity. Recovery (absence of clinical sign such as hyperthermia or local hyperesthesia) occurred between 24 to 48 h post-injection. Analgesics were not used because the cows did not display overt pain manifestations at milking and milk sampling. Anti-inflammatory drugs were not administered since inflammation was the reaction under scrutiny. Cows were milk sampled seven times during the intramammary challenge (see below) and blood samples were taken on five occasions.

**Fig 1:**
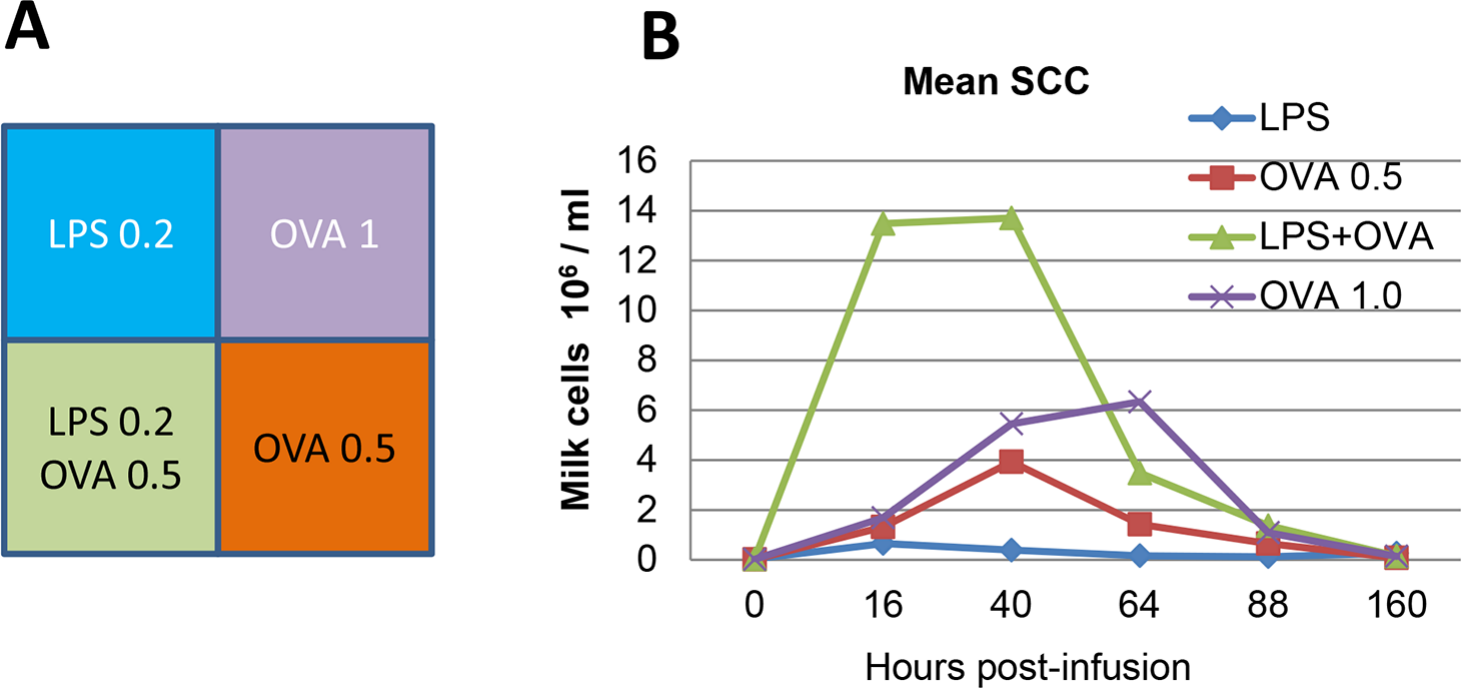
Preliminary experiment to determine the doses of LPS and ovalbumin suitable for induction of synergistic mammary inflammation. A) Schematic representation of the udder with its four quarters and the challenge intramammary infusion. Three cows previously sensitized to ovalbumin received either 0.2 μg LPS, 0.5 or 1 μg ovalbumin, or 0.2 μg LPS and 0.5 μg ovalbumin in combination into one quarter to trigger inflammation. B) Concentrations of milk cells (somatic cell count) in milk of the infused quarters as a function of time post-infusion.

A pilot experiment was designed to determine the doses of LPS or antigen sufficient to trigger a mild inflammatory response evaluated through milk leukocytosis. This experiment involved three cows with four healthy quarters. These cows had been sensitized to OVA and shown to be responders as defined in a previous experiment [18]. Doses of 0.5 or 1.0 μg of pyrogen-free ovalbumin (Calbiochem), 0.2 μg uLPS (ultrapure LPS from *E*. *coli*, devoid of TLR2 agonists, InvivoGen), or the combination of 0.2 μg LPS with 0.5 μg ovalbumin were infused into the lumen of different quarters of each of the three cows through the teat canal with asepsis precautions after the evening milking (Fig 1A). Ovalbumin and LPS were diluted in DPBS supplemented with 0.5 mg/mL pyrogen-free bovine serum albumin (cell culture grade, Sigma) as a vehicle just before use. The quarters were sampled 16, 40, 64, 88 and 160 hours post-infusion (hpi) to determine the somatic cell concentration (SCC) and to check that the glands had not contracted an infection during the follow-up period. Milk samples were taken with asepsis precautions and 50 μL streaked onto a sheep blood agar plate. Cells in milk were counted with an automated cell counter (Fossomatic model 90, Foss Food Technology, Hillerod, Denmark).

For the main experiment, 10 cows were immunized with 50 μg OVA and 50 μg hen egg lysozyme (Sigma) emulsified in Montanide^TM^ ISA 61 VG (Seppic) by the intramuscular route. Two antigens were used to see whether low responder cows to OVA were also low responders to another unrelated antigen. A booster injection was administered 30 days later by using the same regimen except that the antigen dose was 10 μg. The cows were challenged with an intramammary infusion of either 0.5 μg ovalbumin, 0.2 μg LPS, or the combination of 0.5 μg ovalbumin with 0.2 μg LPS into three different healthy quarters. The quarters were sampled before and 8, 16, 40, 64, 88 and 160 hours post-infusion (hpi). In addition to these 10 cows, we included in the immunization schedule four non-responders to ovalbumin identified in a previous experiment [18].

### Quantification of CXCL8, IFN-γ, IL-17A, TNF-α, IL-4 and IL-10 by ELISA

ELISAs for TNF-α, CXCL8 and IL-17A were performed as described previously [17, 19, 20]. IFN-γ and IL-4 concentrations were determined with commercially available bovine IFN-γ or IL-4 ELISA kits (Mabtech AB). Concentrations of IL-10 were determined by using the matched antibody pair for bovine ELISA distributed by AbD Serotec (BioRad) and recombinant bovine IL-10 as standard (Kingfisher Biotech). Concentrations of IL-21 were determined by using the antibodies of the Do-It-Yourself ELISA and the recombinant bovine IL-21 commercialized by Kingfisher Biotech. The lower limits of detection for CXCL8, IFN-γ, TNF-α, IL-10, and IL-21 in milk by the ELISAs were 33, 15, 40, 15 and 200 pg/ml, respectively.

### Whole blood antigenic assay (WBA)

The antigen-specific T cell response was assessed as described [18]. Blood was collected from the caudal blood vessels into commercially available 10-mL evacuated tubes coated with lithium-heparin as anticoagulant (Venosafe™, Terumo® Europe). Samples were used within 4 h after collection. Stimulations were performed in triplicate by mixing 100 μL of blood with either 100 μL of culture medium (RPMI-1640 supplemented with 10% fetal bovine serum, 2 mM L-glutamine, 10 mM HEPES, penicillin-streptomycin, fungizone and 0.05 mM 2-mercaptoethanol) as negative control, 100 μL of pokeweed mitogen (2 μg/mL) as positive control, or 100 μL of ovalbumin or HEL (50 μg/mL) in 96-well microplates (Falcon Microtest™, Becton Dickinson). The culture was incubated at 37°C in a humidified atmosphere with 5% CO_2_ for 3 days. Supernatant were then harvested and stored at -20°C in 96-well plastic storage plates (Greiner™ bio-one) until assayed for cytokine content.

### Statistics

Statistical analyses were performed with the Prism software (GraphPad, version 5.0). Comparisons of paired samples were done with the Friedman’s test followed by Bonferroni post-test comparison when appropriate. Correlation analyses were done with the Spearman’s rank test. A probability level of < 0.05 was considered significant.

## Results

### Synergistic influx of leukocytes into the milk of mammary glands challenged with LPS and ovalbumin

In the preliminary experiment, the doses of either LPS or ovalbumin for the intramammary challenge were based on previous authors’ experience. The aim was to check that the pre-selected doses induced only a mild inflammatory response, and that they were suitable to disclose a potential synergistic inflammatory effect of the LPS-ovalbumin combination. To this end we used three cows that had proven in a previous experiment to be reactive to ovalbumin after sensitization [18]. The monitoring of milk somatic cell count showed that at 16 hpi the inflammatory response to 0.2 μg LPS or to ovalbumin was moderate (Fig 1B). Thereafter, the reaction to ovalbumin increased with a dose-effect that impacted mainly the duration of milk leukocytosis (Fig 1B). The combination of LPS and the smallest dose of ovalbumin tested induced a stronger response essentially at 16 hpi, when the milk leukocytosis was more than additive and thus appeared to be synergistic (Fig 1B). These preliminary results prompted us to conduct another experiment involving more animals.

**Fig 2:**
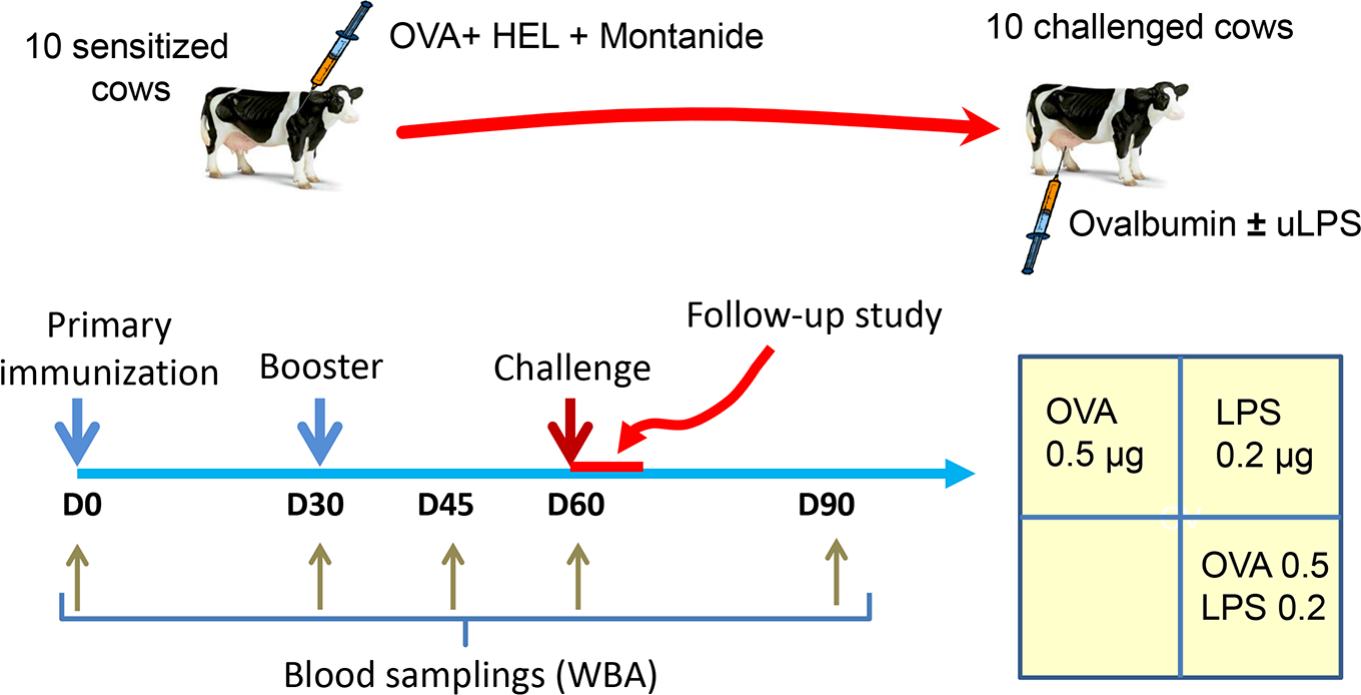
Experimental scheme. Ten lactating cows were immunized by the intramuscular route with ovalbumin (OVA) and hen egg lyzozyme (HEL) emulsified in the Montanide^TM^ ISA 61 VG adjuvant (Seppic) on days 0 and 30. Blood samplings were carried out to monitor the cell-mediated immune response with a whole-blood antigen stimulation assay (WBA). The cows were then challenged by infusion of either OVA, *E*. *coli* LPS, or both agents into the MG through the teat canal on day 60 post-immunization. Milk samples were taken during the follow-up period to monitor the inflammatory response.

**Fig 3:**
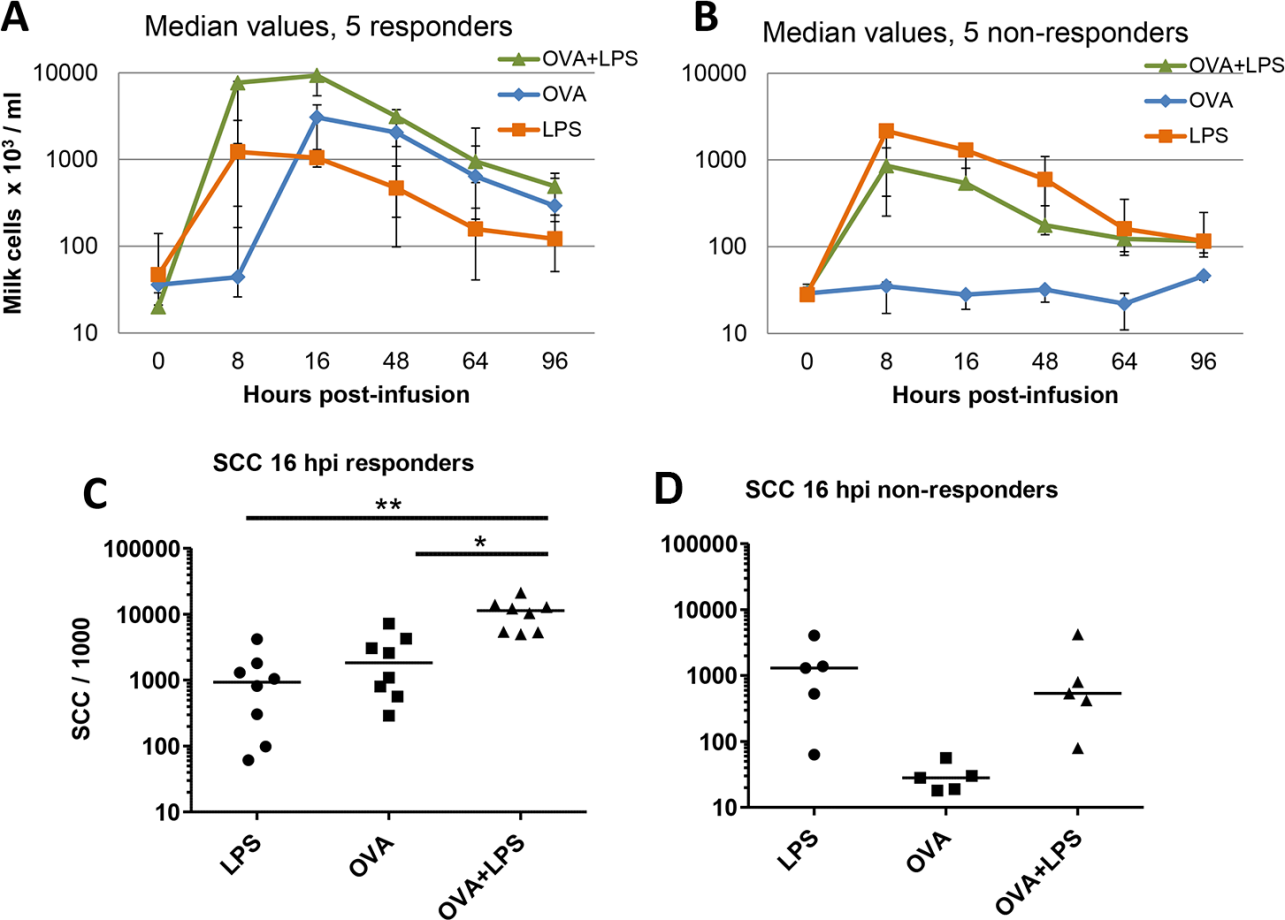
Synergistic effect of innate (LPS) and antigen-specific response to trigger milk leukocytosis in the mammary gland of responder cows previously immunized with ovalbumin. Ten cows immunized with ovalbumin were challenged with either LPS, ovalbumin or both in three different quarters, and milk cell concentrations were monitored. Median values and interquartiles are represented. In quarters of five responder cows that reacted to ovalbumin (A), cell influx was reinforced by the addition of LPS, whereas in the quarters of the five cows that did not respond to ovalbumin, the combination with LPS was without effect (B). C) Concentrations of cells in milk (SCC) at 16 hpi, each point represents one quarter of the eight ovalbumin-responder cows (three cows of the pilot experiment plus five cows of the main experiment). D) Cell concentrations in the milk of the five non-responder cows at 16 hpi. Cell concentrations in milk from quarters infused with ovalbumin and LPS are significantly different from the cell concentrations in milk from quarters infused with either ovalbumin (p < 0.05) or LPS (p < 0.01).

In the main experiment, 10 cows were immunized with OVA and HEL (Fig 2). LPS induced milk leukocytosis in every infused quarter, but only half of the cows reacted to ovalbumin. In samples from the OVA responder cows, increases in milk cell concentrations were not visible at 8 hpi in quarters infused with ovalbumin, a time when SCC concentrations had reached a peak in LPS-infused quarters (Fig 3A). LPS and ovalbumin in combination induced higher cell counts in milk than did LPS or ovalbumin alone, in particular early at 8 hpi (Fig 3A). Cell concentrations at 8 hpi differed significantly (Friedman’s test, p = 0.0008) but due to the limited number of cows in the responder group, the difference between ovalbumin and ovalbumin+ LPS-infused quarters was not significant. By including the data of the preliminary experiment, the differences between ovalbumin-infused and ovalbumin-LPS-infused quarters was significant (p < 0.05; Friedman’s test followed by multiple comparison test with Bonferroni’s correction) (Fig 3B). In quarters of low-responder cows, addition of ovalbumin, which alone did not induce leukocytosis, did not augment the inflammatory response to LPS (Fig 3A).

### Cytokines in milk

**Fig 4:**
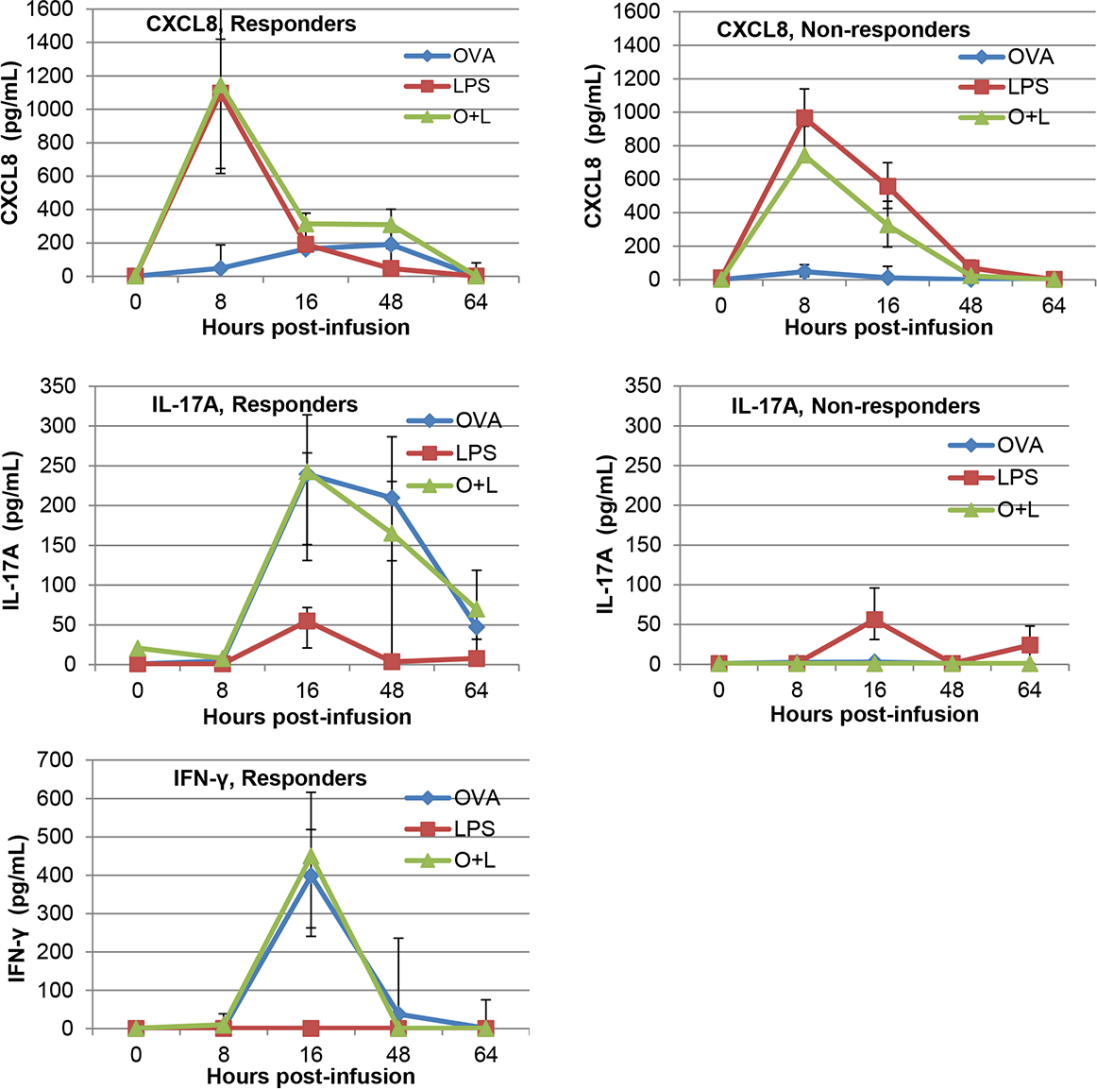
Concentrations of cytokines in milk of quarters infused with either ovalbumin (OVA), LPS or the combination of ovalbumin and LPS (O+L). Data are median values and interquartiles (Q1, Q3) from the five responder cows and the five non-responder cows segregated on the basis of the mammary response to ovalbumin infusion. Concentrations of IFN-γ in the milk of non-responder cows were below the detection level in all the samples (figure not shown).

Concentrations of the chemokine CXCL8 and of the cytokines IL-17A, IFN-γ, TNF-α and IL-10 were assessed in milk samples taken at 0, 8, 16, 48 and 64 hpi from the mammary quarters infused with either 0.2 μg LPS, 0.5 μg OVA, or the combination of these two stimuli. Increases in CXCL8 concentrations coincided with the initial increases in milk cell concentrations in quarters infused with LPS, alone or with OVA (Figs 3A and 4). In the milk of quarters infused with OVA only, there was little cell influx at 8 hpi, and little increase in CXCL8 concentration. The kinetics and magnitude of CXCL8 concentrations were similar in the milk of responder and non-responder cows. CXCL8 concentrations declined sharply as soon as 16 hpi, in contrast with the concentrations of IL-17A and IFN-γ in quarters infused with ovalbumin alone or with LPS, which were low at 8 hpi, peaked at 16 hpi and thus augmented after the onset of milk leukocytosis. These increases occurred only in milk from responder cows, although LPS on its own induced a small increase in IL-17A concentrations at 16 hpi (Fig 4). Concentrations of IFN-γ decreased earlier than IL-17A concentrations and were not found in milk from non-responder cows. At variance with the synergistic effect of LPS and OVA on milk cell concentrations, there was no synergistic increase in IL-17A or IFN-γ concentrations in milk from quarters infused with both agonists compared to concentrations in quarters infused with OVA alone. Concentrations of TNF-α remained below the limit of detection of the ELISA, and IL-10 was found in a few samples at low concentrations (15–45 pg/mL) without clear distribution between treatments and groups (not shown).

### Cell-mediated immune response to ovalbumin

**Fig 5:**
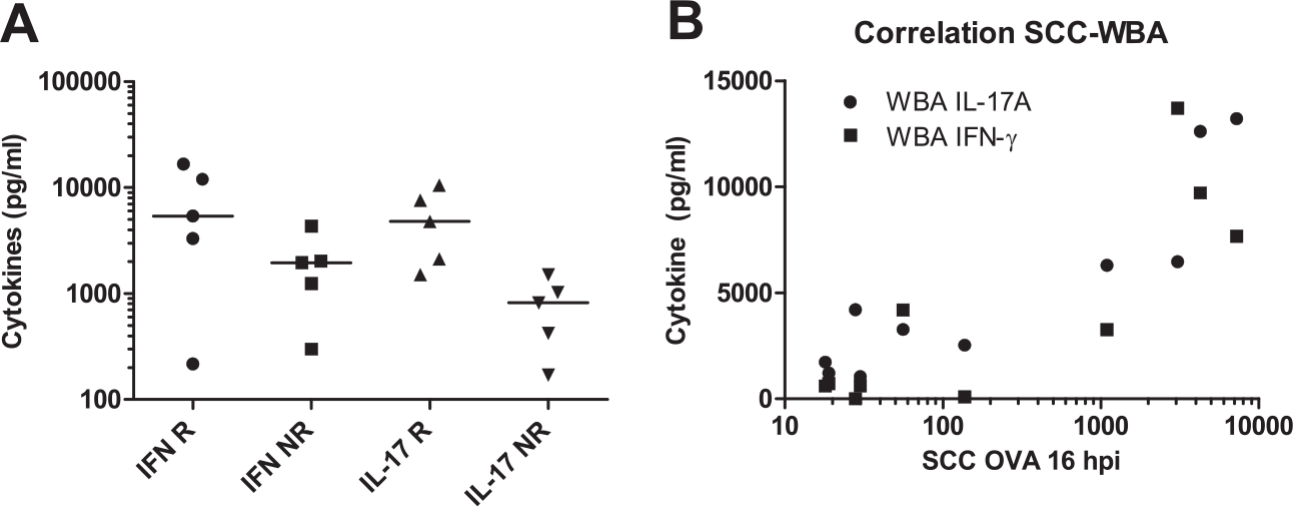
Production of IFN-γ and IL-17A in the OVA-specific WBA and correlation with OVA-specific milk leukocytosis. A) Production of IFN-γ (IFN) and IL-17A (IL-17) in the OVA-specific WBA at 45 days post-first immunization in responder (R) and non-responder cows (NR); B) Correlation between milk leukocytosis at 16 hpi in quarters infused with ovalbumin and production of IL-17A (circles; R = 0.842, P = 0.002) or IFN-γ (squares; R = 0.721, P = 0.019). The figure shows that the circulating leukocytes of cows that did not react to OVA infusion by recruiting leukocytes in the infused quarter (non-responders) produced little concentrations of IL-17A and IFN-γ upon stimulation with ovalbumin.

In a previous study we had found that the ovalbumin-specific production of IL-17A and IFN-γ correlated with the milk leukocytosis induced by the infusion of ovalbumin in the mammary gland of sensitized cows, and thus that the antigen-specific WBA could be used as a predictor of mASR [18]. In the present study also, there was a clear-cut difference in the production of IL-17A, and to a lesser extent IFN-γ, upon OVA stimulation of whole blood of responders and non-responder cows (Fig 5A). Accordingly, the highest correlation was found between IL-17A concentration and SCC in OVA-infused quarters at 16 hpi (Table 1 and Fig 5B). The correlation was also significant between SCC in these quarters and IFN-γ production (Table 1 and Fig 5B). This result confirms our previous results, in support of the suitability of this assay and time of sampling to predict mASR [18]. The correlation was also high between SCC in quarters infused with ovalbumin plus LPS and IL-17A production, but not with IFN-γ production (Table 1). There was no correlation between IL-17A production and SCC in quarters infused with LPS, and a trend toward a negative correlation between IFN-γ production and LPS-induced SCC.

**Table 1:** Correlation matrix established from the IFN-γ or IL-17A production in the ovalbumin-specific WBA performed 45 days after last immunization (D45), and the milk SCC at 8 or 16 hpi in the quarters challenged with ovalbumin (OVA), LPS, or both (O+L).

	IFN-γ D45	IL-17A D45	SCC OVA 8 hpi	SCC OVA 16 hpi	SCC O+L 8 hpi	SCC O+L 16 hpi	SCC LPS 8 hpi	SCC LPS 16 hpi
IFN-γ D45		**0.636**	**-0.055**	0.721*	**-0.079**	**0.358**	**-0.430**	**-0.370**
IL-17A D45	0.054		**0.382**	0.842*	**0.552**	0.770*	**0.067**	**0.103**
SCC OVA 8 hpi	0.892	0.279		**0.539**	**0.503**	**0.333**	**0.309**	**0.382**
SCC OVA 16 hpi	0.023	0.004	0.114		**0.418**	0.721*	**-0.127**	**-0.018**
SCC O+L 8 hpi	0.838	0.105	0.144	0.233		0.830*	0.758*	**0.515**
SCC O+L 16 hpi	0.313	0.013	0.349	0.023	0.005		**0.406**	**0.273**
SCC LPS 8 hpi	0.218	0.865	0.387	0.733	0.015	0.247		0.770*
SCC LPS 16 hpi	0.296	0.785	0.279	0.973	0.133	0.448	0.013	

Data are from the 10 cows of the main experiment. Correlation coefficients (Spearman’s rank test) are in bold, P values in plain figures.

* Significant correlations (P < 0.05)

The correlation between IL-17A and IFN-γ production in the WBA at 45 days post-first immunization was barely significant (R = 0.636; P = 0.054), in accordance with our previous study [18]. Concentrations of IL-4 were below the limit of detection of the ELISA, except for one non-responder cow (230 and 195 pg/mL after stimulation with ovalbumin or HEL, respectively).

Analysis of correlations between milk SCC data shows that in quarters infused with LPS the SCC at 8 and 16 hpi correlated with the SCC in quarters infused with ovalbumin and LPS at 8 hpi but not at 16 hpi (Table 1). This likely occurred because in both LPS and OVA + LPS infused quarters the SCC rose at 8 hpi, whereas at 16 hpi SCC still rose in OVA + LPS infused quarters while SCC had begun to decrease in LPS infused quarters (Fig 3A). There was no correlation between SCC of quarters infused with ovalbumin and SCC of quarters infused with LPS, as expected since the two events involve at least partly distinct mechanisms.

### Relation between cell-mediated immune response to ovalbumin and HEL

**Fig 6:**
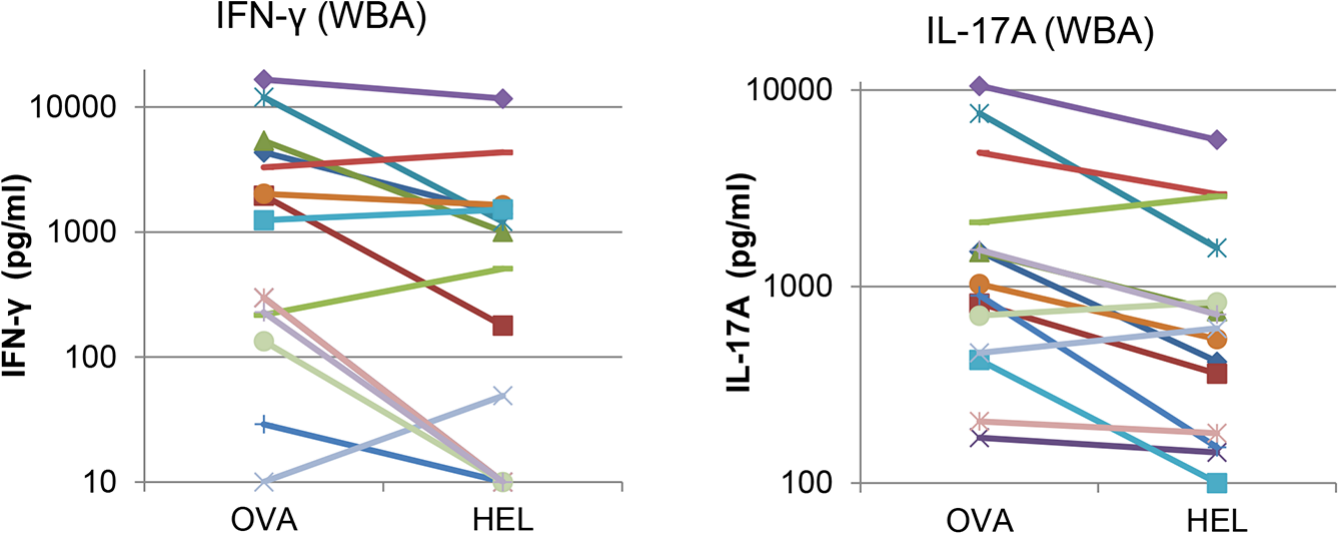
Correlations between the OVA-specific WBA and the HEL-specific WBA. Correlation between immune responses to ovalbumin and HEL in terms of IFN-γ or IL-17A production in the antigen-specific WBA. Data are from the 10 cows of the main experiment and four non-responders to ovalbumin of a previous study, immunized with OVA and HEL concurrently with the other 10 cows.

In our previous studies we had been surprised by the proportion of low or non-responders to ovalbumin, which was about one out of 3 cows [17, 18]. This proportion was even higher in the present study, reaching 50% of the immunized cows. We hypothesized that the low immune response to ovalbumin could result from a “gap” in the MHC class II repertoire. In a preliminary attempt to test this hypothesis, we performed immunization with a mixture of ovalbumin and HEL, supposing that the immune response to these distant antigens would be unrelated. Contrary to this supposition, there was a significant correlation between the production of IFN-γ in the WBA after stimulation with OVA or HEL (R = 0.745, P = 0.017), and also between the IL-17A production in response to the two antigens (R = 0.685, P = 0.035). Furthermore, the four low-responder cows to OVA available from our previous study that were immunized with HEL were also low responders to HEL, as the production of IL-17A and IFN-γ upon restimulation with HEL was in the range of the production of the non-responders of the present study (Fig 6). These results do not support the MHC class II “gap” hypothesis.

### The response to LPS did not correlate with the response to ovalbumin

**Fig 7:**
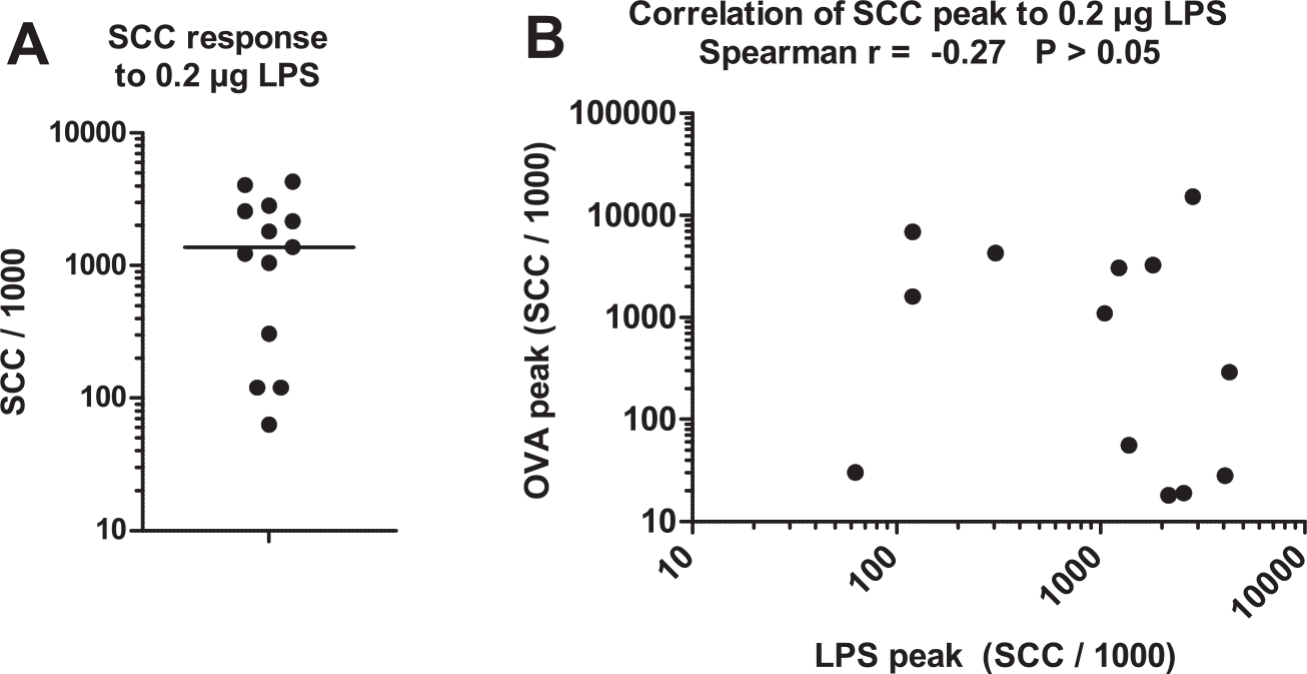
The milk leukocytosis response to a low dose of LPS distinguished high and low responders to LPS, without correlation with the response to ovalbumin. A) Peak SCC response to the infusion of a low dose (0.2 μg) of *E*. *coli* LPS into one quarter of the MG. B) Correlation between SCC peak values in quarters infused with either LPS or OVA. Data are from the three cows of the preliminary experiment and the 10 cows of the main experiment.

With the relatively low dose of LPS used in this study, there was a clear-cut segregation between high and low responders to intramammary infusion of LPS (Fig 7). Milk leukocytosis induced by LPS or ovalbumin did not correlate (Fig 6B), in line with the assumption that the reactivity to these two agonists was not under the same determinism.

## Discussion

Attempts to develop vaccines against mastitis have primarily been focused on the induction of antibodies to bacteria and bacterial components [2, 21–23]. Yet, it has long been known that cell-mediated immune responses contribute to the manifestations of mastitis [24, 25]. Recently, there has been a renewed interest in immune responses of the MG mediated by CD4+ or CD8+ T lymphocytes [12, 13, 17, 26]. We showed that CD4+ T cells were likely involved in the antigen-specific neutrophilic inflammation triggered by the exposure of the MG to minute amounts of a sensitizing antigen [18]. In the event of an infection, the MG is exposed to both MAMPs and bacterial antigens. This raises the issue of a possible synergy between innate and adaptive inflammatory responses in the MG.

The main objective of this study was to investigate whether the innate immune response triggered by MAMPs can be modulated by the adaptive immune response to a sensitizing antigen in the MG. The MG is sensitive to MAMPs such as LPS, lipoteichoic acid, peptidoglycan subunits and lipoproteins [27–30]. Mammary epithelial cells are likely to contribute to setting off the ensuing inflammatory response through their capacity to sense and react to a variety of MAMPs [27, 31–34]. Mammary epithelial cells are also able to react to IL-17A and IL-17F, in particular in the presence of MAMPs [11]. We speculated that this in vitro synergy could be paralleled in vivo by the synergy between the innate reaction to MAMPs and an antigen-specific inflammatory response. The synergistic response of quarters infused with both LPS and ovalbumin displayed in our experiments strongly suggests that innate and adaptive responses can synergize in the bovine MG. Specifically, mammary ASR amplified the early response of the MG to LPS (Fig 3). The contribution of an adaptive immune response was supported by the fact that only cows responding to ovalbumin displayed the synergy with LPS. The magnitude of the synergistic milk leukocytosis correlated with the production of IL-17A and IFN-γ in milk and in vitro in the antigen-specific WBA, which provides circumstantial evidence that these two cytokines and the cells that produce them are behind the observed synergy. As IL-17A and IFN-γ released in the ovalbumin-specific WBA depend on the presence of CD4+ T cells [18], it is plausible that Th17 and possibly Th1 cells contributed to the synergy.

It is of interest that in the milk of responder cows, the synergy between responses to LPS and ovalbumin was manifest at 8 hpi, when the response to ovalbumin alone was not detected by the SCC or CXCL8 or cytokine concentration increases. This suggests that the events behind the synergy, including the antigen-specific response, had been in effect before 8 hpi. These events took place in the mammary tissue and thus escaped the monitoring of milk parameters. What was measured in milk could rather be the consequence, not the cause, of the inflammatory response. An example is CXCL8, which can be produced by MEC but also by recruited leukocytes [35], concentrations of which were not tightly related to milk cellular influx and not modified by the antigen-specific local response even in responder cows (Figs 3 and 4). Also, elevated concentrations of IL-17A and IFN-γ were not detected at 8 hpi, a time when the peak of SCC was already reached (Figs 3 and 4). This suggests that these cytokines were produced in the mammary tissue by resident T cells but not in milk at this early time. Alternatively, or concurrently, cytokines other than those measured in this study could have been involved in milk leukocytosis.

In this study, a dose of 0.5 μg OVA induced sizeable increases in IL-17A and IFN-γ concentrations in milk. Previously we reported comparable concentrations of these two cytokines following intramammary infusion of higher amounts (10 to 25 μg) of ovalbumin in the milk of sensitized responder cows [17, 18]. A dose-effect was nonetheless obvious on the kinetics of cell recruitment into milk, which was delayed by a few hours with the low dose of OVA. This dose-effect on the lag phase preceding milk leukocytosis had been already been observed in cows sensitized to staphylococcal alpha toxin [16].

A secondary objective of this study was to test whether the high proportion of non-responder cows is specific to this antigen or whether it is a more general phenomenon. Co-immunization with ovalbumin and HEL led also to a sizeable proportion of low antigen-specific WBA responders. There was a high correlation between the responses to ovalbumin and HEL. This tends to rule out the MHC class II gap hypothesis, although the limited number of antigens and animals calls for caution. The non-responder issue could be of importance if it occurs also with more complex vaccine preparations. Whatever it may be, it appears that the antigen-specific WBA is instrumental in detecting non-responders, and a better understanding of its underlying mechanism could constitute a lead to decipher the reasons behind the absence of response. Delayed-type hypersensitivity is under genetic control and has been shown to be heritable in cattle [36], which opens up the possibility of genetic selection for high or low responder cows [37].

Besides non-responders to ovalbumin and HEL, there were also low responders to LPS among the 14 cows under experiment. The use of a low dose of LPS was probably instrumental in revealing the inter-animal difference in susceptibility. There was no correlation between the response to LPS and the response to ovalbumin in terms of milk leukocytosis, which is not surprising since the two agonists are supposed to act through innate and adaptive arms of the immune system, respectively. There was even a trend for a negative correlation between SCC induced by LPS at 8 hpi and 16 hpi and IFN-γ concentrations in the WBA (Table 1). There was also a slight negative correlation between SCC values at 8 hpi in quarters infused with LPS and SCC values at 16 hpi in quarters infused with OVA (Table 1). An inter-animal difference in susceptibility of bovine dermal fibroblasts to LPS has been reported and related to the outcome of experimentally induced *E*. *coli* mastitis, the low responder cows faring better than the high responders [38]. This individual variability was shown to depend at least in part on epigenetic regulation [39]. It would be of interest to investigate the impact of this variable response to LPS on the outcome of *E*. *coli* mastitis.

In conclusion, this study shows that the innate immune response to MAMPs can synergize with the adaptive immune response to a protein antigen, resulting in an amplified milk leukocytosis at the onset of the inflammatory reaction. Enhancement of the innate immune response to bacteria by adaptive immunity is effective at the start of the inflammatory response. The magnitude of the cell recruitment is likely to be associated with qualitative modifications of the immune response, with an impact on the activities of the recruited cells, and also of the resident cells such as mammary epithelial cells. Our knowledge of these modifications is limited but we know that neutrophil bactericidal activity is augmented [16] and that the IL-17A produced locally at the epithelial barrier during mASR modifies the response of mammary epithelial cells to bacteria or bacterial components [7, 11]. At the onset of infection, the MG is confronted with bacteria that release both MAMPs and antigens. Thus the stage is set for a synergistic innate and adaptive response in animals sensitized through previous encounters with the antigens. The consequences of this synergistic response in terms of severity, duration, and sequels of intramammary infections are poorly appreciated at the moment. This prompts further studies on this line of research. The novel model of mammary inflammation presented in this study, which associates stimuli of the innate and adaptive immune systems, has a promising potential with a view to investigating vaccine immune responses and to contributing to the development of mastitis vaccines. Less aggressive to experimental animals, it can be used as a preliminary or surrogate to experimental infections.
